# Effectiveness of an 8-Week Physical Activity Intervention Involving Wearable Activity Trackers and an eHealth App: Mixed Methods Study

**DOI:** 10.2196/37348

**Published:** 2022-05-03

**Authors:** Gavin R McCormack, Jennie Petersen, Dalia Ghoneim, Anita Blackstaffe, Calli Naish, Patricia K Doyle-Baker

**Affiliations:** 1 Department of Community Health Sciences Cumming School of Medicine University of Calgary Calgary, AB Canada; 2 Faculty of Sport Sciences Waseda University Tokyo Japan; 3 Faculty of Kinesiology University of Calgary Calgary, AB Canada; 4 School of Planning, Architecture, and Landscape University of Calgary Calgary, AB Canada; 5 Faculty of Applied Health Sciences Brock University St Catharines, ON Canada; 6 Department of Communication, Media and Film Faculty of Arts University of Calgary Calgary, AB Canada; 7 Alberta Children’s Hospital Research Institute University of Calgary Calgary, AB Canada

**Keywords:** activity tracker, technology, eHealth, physical activity, intervention, exercise, mHealth, fitness, wearable, sensor, digital health, COVID-19, health promotion, mixed methods study, wearable technology

## Abstract

**Background:**

Health-promotion interventions incorporating wearable technology or eHealth apps can encourage participants to self-monitor and modify their physical activity and sedentary behavior. In 2020, a Calgary (Alberta, Canada) recreational facility developed and implemented a health-promotion intervention (Vivo Play Scientist program) that provided a commercially available wearable activity tracker and a customized eHealth dashboard to participants free of cost.

**Objective:**

The aim of this study was to independently evaluate the effectiveness of the Vivo Play Scientist program for modifying physical activity and sedentary behavior during the initial 8 weeks of the piloted intervention.

**Methods:**

Our concurrent mixed methods study included a single-arm repeated-measures quasiexperiment and semistructured interviews. Among the 318 eligible participants (≥18 years of age) registered for the program, 87 completed three self-administered online surveys (baseline, T_0_; 4 weeks, T_1_; and 8 weeks, T_2_). The survey captured physical activity, sedentary behavior, use of wearable technology and eHealth apps, and sociodemographic characteristics. Twenty-three participants were recruited using maximal-variation sampling and completed telephone-administered semistructured interviews regarding their program experiences. Self-reported physical activity and sedentary behavior outcomes were statistically compared among the three time points using Friedman tests. Thematic analysis was used to analyze the interview data.

**Results:**

The mean age of participants was 39.8 (SD 7.4) years and 75% (65/87) were women. Approximately half of all participants had previously used wearable technology (40/87, 46%) or an eHealth app (43/87, 49%) prior to the intervention. On average, participants reported wearing the activity tracker (Garmin Vivofit4) for 6.4 (SD 1.7) days in the past week at T_1_ and for 6.0 (SD 2.2) days in the past week at T_2_. On average, participants reported using the dashboard for 1.6 (SD 2.1) days in the past week at T_1_ and for 1.0 (SD 1.8) day in the past week at T_2_. The mean time spent walking at 8 weeks was significantly higher compared with that at baseline (T_0_ 180.34 vs T_2_ 253.79 minutes/week, *P*=.005), with no significant differences for other physical activity outcomes. Compared to that at baseline, the mean time spent sitting was significantly lower at 4 weeks (T_0_ 334.26 vs T_1_ 260.46 minutes/day, *P*<.001) and 8 weeks (T_0_ 334.26 vs T_2_ 267.13 minutes/day, *P*<.001). Significant differences in physical activity and sitting between time points were found among subgroups based on the household composition, history of wearable technology use, and history of eHealth app use. Participants described how wearing the Vivofit4 device was beneficial in helping them to modify physical activity and sedentary behavior. The social support, as a result of multiple members of the same household participating in the program, motivated changes in physical activity. Participants experienced improvements in their mental, physical, and social health.

**Conclusions:**

Providing individuals with free-of-cost commercially available wearable technology and an eHealth app has the potential to support increases in physical activity and reduce sedentary behavior in the short term, even under COVID-19 public health restrictions.

## Introduction

### Background

Daily participation in physical activity provides numerous benefits, including enhancing physical and mental health and reducing the risk of chronic disease [1]. Despite these benefits, individuals report barriers to maintaining regular physical activity routines such as lack of time, confidence, and money, and unsupportive physical and social environments [2-5]. However, physical activity interventions offer individuals opportunities to initiate, maintain, and modify their physical activity routines [6,7]. Moreover, evidence suggests that sedentary behavior (eg, sitting) can negatively impact health independent of physical activity levels [8,9]. Sedentary behavior is defined as any waking behavior that has a relative energy expenditure ≤1.5 metabolic equivalents (METs) while in a sitting, reclining, or lying posture [10]. Physical activity interventions with and without specific components that target sedentary behavior can reduce sedentary time [11]. The current Canadian 24-Hour Movement Guidelines recommend accumulating sufficient physical activity, reducing prolonged sitting and sedentary behavior, as well as obtaining adequate sleep regularly, regardless of age [12,13].

Physical activity interventions incorporating wearable activity trackers (eg, smart watches, fitness trackers, and pedometers) that allow users to quantify self-movement offer wearers immediate behavioral feedback and movement data that can support them in modifying current or future physical activity levels [14-17]. This evidence is encouraging given the growth in the popularity of commercial wearable trackers among consumers for monitoring physical activity and fitness [16,18-21]. Moreover, wearable trackers are often coupled with eHealth (including mobile health) apps on smartphones or other screened devices that provide users with complementary detailed information about behavior patterns (eg, duration and intensity of physical activity, sedentary time, sleep, and energy expenditure), biometrics (eg, heart rate, blood oxygen saturation, and body temperature), and geographical location or global positioning [21,22]. Data from wearable trackers and eHealth apps can support the setting and achievement of behavioral goals, facilitate social comparison or competition, and incorporate individual- or group-based activity through synchronous or asynchronous behavioral challenges. Wearable trackers and eHealth apps can also provide automated personalized health-promotion messages or movement notifications that motivate or nudge users to undertake more physical activity or less sedentary behavior [16,17,23-26].

Interventions involving the use of commercially available wearable activity trackers have found positive effects on physical activity [15,17,24,26,27] and weight status [26,28] among adults, including clinical and healthy populations [15,17,27]. Moreover, wearable activity trackers and eHealth apps can reduce sedentary time [29]. Wearable activity trackers can encourage immediate, synchronous changes in physical activity and sedentary behavior [17,24]. Barwais et al [24] found that even over a short period (ie, 4 weeks), participants enrolled in an intervention incorporating the daily use of a wearable activity tracker and receipt of personalized device–informed messaging and prompts significantly increased their volume of walking; increased light-, moderate-, and vigorous-intensity physical activity; and reduced their sedentary time. The most effective physical activity interventions involving wearable technology may include those that incorporate concurrent use of a wearable activity tracker and an eHealth app [17].

### Vivo Play Scientist Program

Community-based health-promotion programming provides a vital and cost-effective strategy for increasing physical activity [30] because of the ability to reach large and diverse populations. Recreational facilities that engage in this type of programming typically include diverse offerings of activities and services, and are therefore well-positioned to deliver physical activity programs to their surrounding catchment communities. Our study focuses on an evaluation of what could be considered an innovative community-based health-promotion program, *The Vivo Play Scientist (VPS) program*, offered by a large North Central Calgary recreational center with approximately 6500 members (Vivo for Healthier Generations). Vivo, established in 2004, is a charity that operates the Centre for Well-being and Innovation lab. Vivo offers a mix of family-based and community-delivered physical activity and play programming to surrounding neighborhoods (eg, park-based play events and take-home play kits). Between November 2020 and March 2021, the VPS program was piloted and independently evaluated by our team. Vivo developed and implemented the VPS program with the aim of increasing physical activity and reducing sedentary behavior. The program involved providing participants, free of charge, with a wearable tracker with integrated syncing technology (Garmin Vivofit4) and access to an eHealth dashboard to support self-monitoring of physical activity and sedentary behavior. The program strategy appeared to align with social cognitive theory [31,32] and control theory [33], both of which recognize the importance of self-monitoring and progress feedback to inform behavior modification and reinforce monitored behaviors. The VPS program was implemented under COVID-19 public health restrictions. The public health restrictions in place during the VPS program included social gathering limits in private and public spaces and occupant capacity limits for businesses. Moreover, recreational facilities, including indoor children’s play centers and indoor playgrounds and fitness and sports facilities, were closed from November 27, 2020, to March 8, 2021. Mandatory masking and physical distancing were also in effect throughout the program and working from home was recommended.

**Figure 1:**
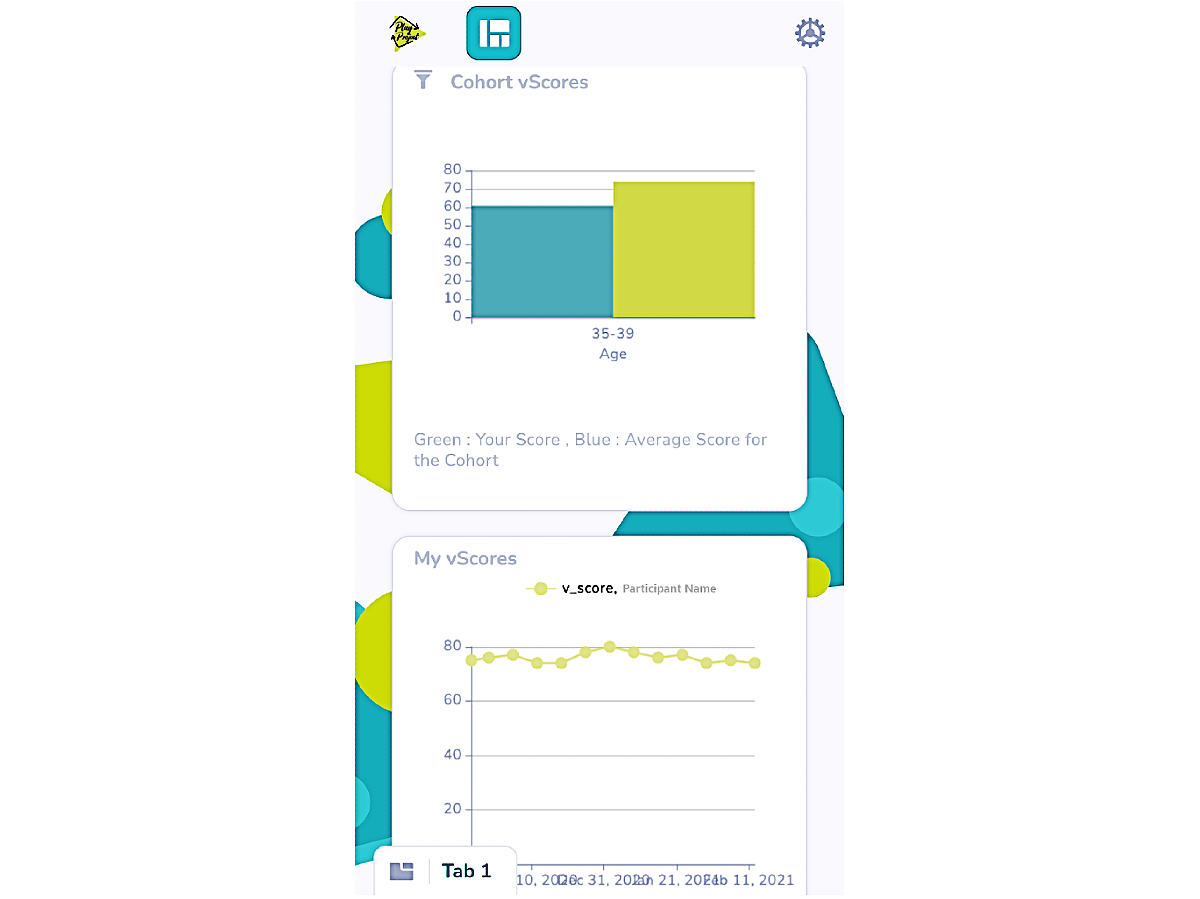
Screenshot of the dashboard information available to Vivo Play Scientist program participants.

The VPS program was advertised on the Vivo website, social media (Facebook and Twitter), and via an email list of clientele affiliated with the facility. The program offered volunteering individuals and families (with a child aged 5-17 years) with a wrist-worn activity tracker (Garmin Vivofit4; approximate value US $80) and access to a customized eHealth dashboard at no financial cost to participants. Participants also had access to the Garmin Connect platform for processing the data captured by the Vivofit4 accelerometer, which provides summaries of physical activity and other metrics (ie, step count, distance travelled, intensity minutes, energy expenditure, and sleep). This platform also offers opt-in physical activity and step challenges, and provides options for setting personalized step goals. Vivofit4 includes a function that over time automatically monitors activity levels and assigns a daily step goal, and a function that notifies the wearer to move after an extended period of inactivity. The customized Vivo eHealth dashboard was developed by White Whale Analytics [34] and provided the health analytics platform, which incorporated a score (ie, vScore Health) supported by Vivametrica data [35]. The vScore is derived from a proprietary algorithm that combines the step count from Vivofit4 along with the participant’s age, sex, height, and weight. Higher vScores [35] reflect better health, and the dashboard provided sex- and age-informed normative values. Participants could share and compare their vScores with other household members participating in the program. On the dashboard, participants could view their current week’s vScore, a historical trend of vScores for each member of their family as a line chart, as well as a comparative bar chart of their vScore to that of their age group within the cohort (see Figure 1). The vScores range between 0 and 100, and include health rankings based on cut-off points (poor, 0-49; fair, 60-62; good, 63-73; very good, 74-85; excellent, 86-100). Although the vScore appears to have face validity, there is limited evidence pertaining to its predictive validity in relation to behavior change and health outcomes. A 12-week intervention involving a small clinical sample of middle-aged women (N=36) that included the use of a wearable activity tracker and access to Vivametrica performance data found a median increase of 9% in daily step counts; however, this change was not statistically significant [36]. The authors (including the founder of Vivametrica) reported that 28% of the participants did not access the Vivametrica output data and the majority of those who accessed this information did so fewer than five times during the intervention [36]. Vivo hypothesized that the access to behavior feedback from the eHealth dashboard, Garmin Connect, and Vivofit4 would motivate participants to modify their physical activity and sedentary behavior.

Participants who registered for the program received written instructions and attended an on-boarding video conference call regarding the use and syncing of Vivofit4, Garmin Connect, and the eHealth dashboard, along with information to assist them in interpreting the vScore. Participants were encouraged to wear Vivofit4 during all waking hours and to synchronize Vivofit4 with Garmin Connect and the dashboard once per week. However, participants did not receive instructions on how often they should access Garmin Connect or the dashboard and what information or outputs they should consult, nor did they receive any prescriptive behavioral goals or targets to achieve in relation to physical activity or sedentary behavior. Participants could use or explore all or any functions within Vivofit4, Garmin Connect, or the dashboard (eg, setting up movement reminders, signing up for challenges, joining activity communities, sharing activity progress, sharing vScores, and tracking sleep). The intention of the VPS program was to offer access to the wearable tracker and eHealth dashboard, and allow participants autonomy in deciding how best to use these tools to support their own physical activity goals.

### Study Aim

This study is part of a larger research project evaluating the feasibility and effectiveness of the VPS program. A concurrent mixed methods research approach was used to evaluate the effectiveness of the VPS program for modifying physical activity and sedentary behavior in the initial 8 weeks of its implementation.

## Methods

### Ethics Approval

The University of Calgary Conjoint Health Research Ethics Board approved the study (REB20-1218).

### Study Design and Recruitment

We undertook a concurrent mixed methods single-arm repeated-measures design with semistructured interviews. Volunteers were screened for eligibility and registered in the VPS program with the assistance of Vivo staff. Eligible participants had access to the internet, resided in a North Central Calgary neighborhood, were ≥18 years of age, and had a current email address. Multiple members of a single household could participate in the VPS program; however, only one self-selecting adult per household could participate in the program evaluation. Eligible registered participants (N=318) were sent a recruitment email with study information, consent form, web link to a baseline online questionnaire, and identification number to access the online questionnaire. Among those sent a recruitment email, 153 participants completed the baseline questionnaire. A subset of participants were invited to undertake a postprogram semistructured interview via telephone or video conference. The semistructured interviews provided supporting data for the quantitative results [37]. To capture a range of different perspectives and a sample that was diverse in age, gender, ethnicity, education level, employment, and income characteristics, we recruited the interview participants using a maximum-variation sampling approach [38]. The interview sample included those who had and had not completed the 8-week VPS program.

### Data Collection

The online questionnaires were delivered using the Qualtrics platform (Toronto, Canada) and were administered at baseline (T_0_), 4 weeks (T_1_), and 8 weeks (T_2_). To increase compliance, for each questionnaire completed, participants received entry into a prize draw to win one of two CAD $500 (~US $400) gift cards. The questionnaires captured information, including sociodemographic characteristics, perceptions and use of wearable technology and eHealth apps, physical activity cognitions (eg, attitudes, self-efficacy, perceived barriers and benefits), and self-reported physical activity and sedentary behavior. This study included a subset of variables from the questionnaires, specifically self-reported physical activity and sedentary behavior, history of using wearable technology and eHealth apps, and sociodemographic characteristics. Each questionnaire took 20-30 minutes to complete.

The telephone-based semistructured interviews were 30-45 minutes in duration. Participants who completed the interview received a CAD $25 (~US $20) gift card as a token of appreciation. An interviewer, trained in qualitative research methods, asked several open-ended questions regarding participant experiences of the VPS program, including the use of Vivofit4, Garmin Connect, and the eHealth dashboard; the effect of the program on their physical activity and health; and recommendations for improving the program. Given our interest in exploring the effectiveness of the program for modifying physical activity and sedentary behavior, we focused on responses to the following six interview questions: *Which features of the Vivofit4 were most or least useful to you in supporting your physical activity? Which features of Garmin Connect were most or least useful to you in supporting your physical activity? Which features of Vivo Play Scientist Health Dashboard were most or least useful to you in supporting your physical activity? What have you discovered about your physical activity and health as a result of participating in the program? Have you noticed any changes in your behavior since the start of the program? How has your usage of the device changed since you first received it?*

### Quantitative Variables

#### Frequency of Using Vivofit4 and the eHealth Dashboard

At the 4-week (T_1_) and 8-week (T_2_) surveys, participants reported how many days in the past week and the usual amount of time per day they had used Vivofit4 and the dashboard. In addition to reporting days of VivoFit4 wear and dashboard use as continuous outcomes (both of which had skewed distributions), we categorized Vivofit4 use to capture participants who had used the wearable activity tracker on most days (ie, ≥4 days/week vs <4 days/week). Data presented in the dashboard were automatically updated weekly; therefore, we categorized frequency of use into ≥1 day/week versus <1 day/week.

#### Physical Activity

At the baseline (T_0_), 4-week (T_1_), and 8-week (T_2_) surveys, physical activity was measured using the International Physical Activity Questionnaire Short-Form (IPAQ-SF). The IPAQ-SF has acceptable reliability and validity [39]. Minutes of walking, moderate-intensity physical activity (MPA), and vigorous-intensity physical activity (VPA) in the past week were captured. We applied the IPAQ-SF scoring protocol to correct for overreporting of physical activity minutes and to estimate total weekly physical activity incorporating the relative intensity (MET) of each activity (ie, MET [minutes/week]=[VPA minutes×8 METs]+[MPA minutes×4 METs]+[walking minutes×3.3 METs]) [40,41]. Accumulating 30 minutes of moderate-to-vigorous physical activity (MVPA) most days provides health benefits in adults [42]. In each survey (T_0_, T_1_, and T_2_), a single item captured the number of days in the past week that the participant accumulated at least 30 minutes of MVPA (ie, sport or exercise, brisk walking or cycling for recreation, or to get to and from places, but excluding occupational activity and housework) [43]. This measure of sufficient MVPA has acceptable test-retest reliability and concurrent validity in relation to other single-item measures of physical activity [43].

#### Sedentary Behavior

Two items captured sedentary behavior at baseline (T_0_), 4 weeks (T_1_), and 8 weeks (T_2_). One item from the IPAQ-SF measured the usual time spent sitting (ie, at work, at home, during course work, traveling by motor vehicle, and for leisure) in the last 7 days [39]. Another item captured leisure-based screen time: “In an average week, how much time per day do you usually spend watching television or other screen-based electronic devices outside your workplace (eg, video games, computer games, DVD/movies, internet, email, texting, smartphone)?” A similar item has been used previously to measure leisure-based screen time in Canadian adults [44-46]. We modified the item to also capture contemporary sedentary activities (eg, use of mobile technology).

#### Sociodemographic Characteristics

Sociodemographic characteristics, including age, sex, ethnicity, household income, employment status, education, marital status, number of dependents in the household, dog ownership, and composition of household members participating in the intervention (ie, one adult only, multiple adults only, one adult and children, or multiple adults and children), were also collected at baseline (T_0_).

### Analysis

Descriptive statistics (means, standard deviations, and frequencies) were calculated for the sample characteristics. Most outcome variables were nonnormally distributed (positively skewed); therefore, we applied nonparametric statistical tests. All physical activity (weekly minutes of walking, MPA, VPA, total physical activity, and sufficient daily MVPA) and sedentary behavior (daily minutes of sitting and screen time) outcomes were analyzed using the Friedman test to compare the differences in mean rank across the three time points (T_0_, T_1_, and T_2_). Using the significant results in the Friedman tests (*P*<.05), we undertook a priori comparisons employing Wilcoxon signed-rank tests to identify statistically significant differences in outcomes between time points relative to baseline (ie, T_0_ vs T_1_ and T_0_ vs T_2_). To reduce the chance of type 1 error, pairwise differences from the planned comparisons were considered statistically significant based on an adjusted *P*<.025. In addition, we assessed for effect modification by comparing gain scores (ie, T_2_–T_0_) for physical activity and sedentary behavior outcomes between subgroups (ie, adults-only participating households vs households with child participants; ever used vs never used a wearable tracker; and ever used vs never used an eHealth app) using Mann-Whitney *U* tests and stratified analysis (Friedman tests with Wilcoxon signed-rank tests for planned comparisons) to determine if group responses to the intervention were heterogeneous from the beginning (T_0_) and to the end of the intervention (T_2_). Quantitative analysis was performed using SPSS version 24.

Audio data collected during the semistructured interviews were transcribed verbatim and analyzed using thematic analysis [47]. Qualitative data were organized and analyzed using NVivo version 12. Three researchers (JP, DG, and PKDB) coded the data and identified the themes. Member checking, peer review, and an audit trail were employed as strategies to enhance the trustworthiness of the qualitative results. Triangulation of the quantitative and qualitative results was undertaken during the interpretative phase of the findings.

## Results

### Sample Characteristics

**Figure 2:**
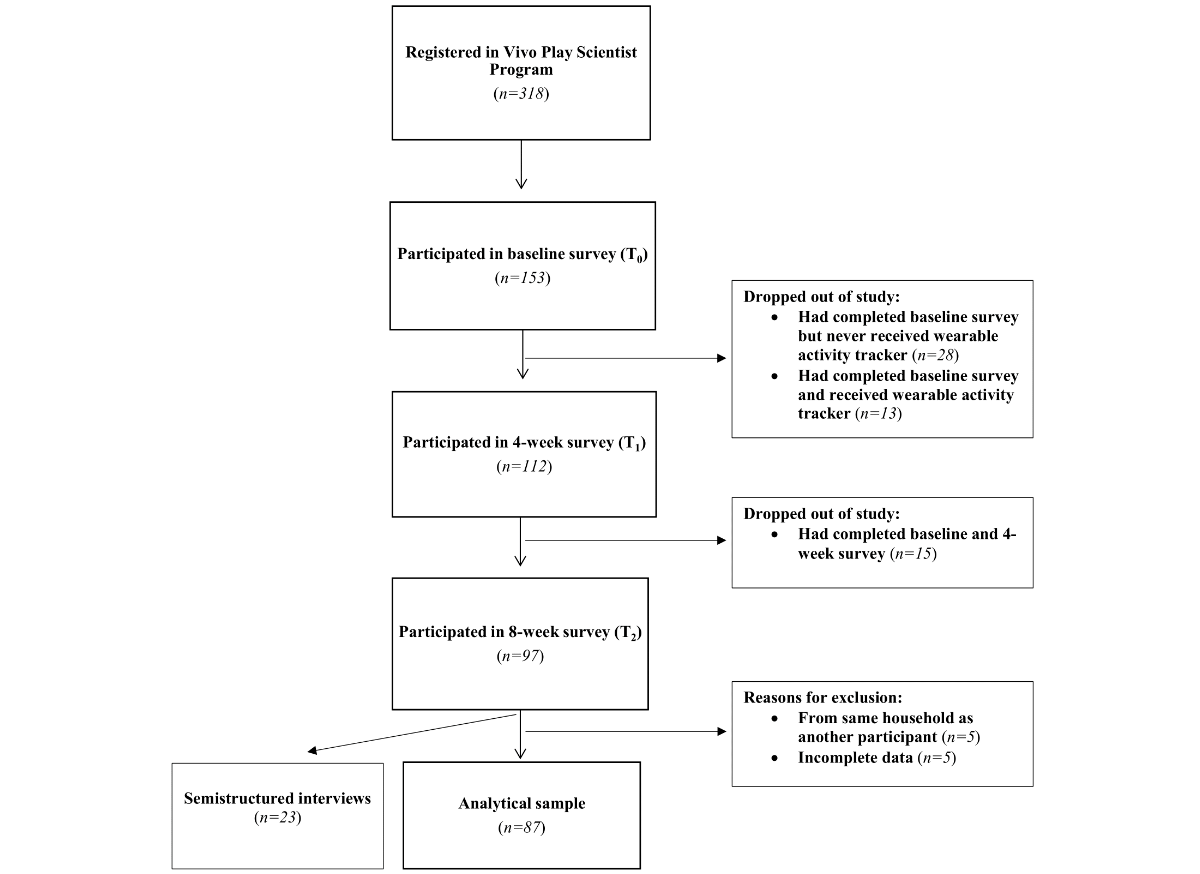
Flow diagram of participant recruitment.

The flow of participants through the study is shown in Figure 2. The analytical sample included 87 participants with complete data for all three surveys (T_0_, T_1_, and T_2_). Excluded participants (n=66) did not participate in all three surveys, had incomplete data, or were members of the same household. Sociodemographic characteristics and baseline physical activity and sedentary behavior were similar between the analytical sample and excluded cases, with the exception that excluded cases had a lower proportion of participants reporting an income of CAD $80,000-119,999 (US $65,000-94,999) per year (13.6% vs 37.9%, *P*<.001) and a higher proportion reporting they did not know or refused to answer (28.8% vs 10.3%, *P*=.003). The semistructured interviews were conducted with 23 participants (18 women, 5 men; aged 22-56 years).

Our sample consisted mostly of participants who reported being female, having a university education, being non-Caucasian, working full or part time, married or common-law, and not owning a dog (Table 1). Over one-quarter of households had gross annual incomes of at least CAD $119,999 (US $94,999)/year. The mean age of participants was 39.4 years and the mean number of children <18 years of age in the home was 1.8.

Almost half of all participants reported prior use of a wearable tracker (40/87, 46%) or eHealth app (43/87, 49%). On average, participants reported wearing Vivofit4 for 6.3 (SD 1.7) days in the past week at T_1_ and for 6.0 (SD 2.2) days in the past week at T_2_. Most participants reported using Vivofit4 ≥4 days/week (T_1_: 93.1% [n=81/87] and T_2_: 87.4% [n=76/87]). On average, participants reported usually wearing Vivofit4 for approximately 12.5 hours/day at T_1_ (mean 751.2, SD 201.7 minutes) and T_2_ (mean 756.8, SD 211.9 minutes). The median usual wear time was 840 minutes/day at both T_1_ and T_2_. On average, participants reported using the eHealth dashboard for 1.6 (SD 2.1) days in the past week at T_1_ and for 1.0 (SD 1.8) day in the past week at T_2_. Approximately half of the participants reported using the dashboard ≥1 day/week (T_1_: 54.0% [n=47/87] and T_2_: 47.1% [n=41/87]). Approximately two-thirds of participants were from households where at least one adult and one child participated in the program with the remainder being from households with no children participating (Table 1).

**Table 1:** Baseline characteristics of intervention participants (N=87).

Characteristic			Value
**Sex, n (%)**			
	Male	22 (25)	
	Female	65 (75)	
**Education, n (%)**			
	No university	25 (29)	
	Completed university	62 (71)	
**Annual household income (CAD $^a^), n (%)**			
	<80,000	22 (25)	
	80,000-119,999	33 (38)	
	>119,999	23 (26)	
	Don’t know/refuse to answer	9 (10)	
**Ethnicity, n (%)**			
	Chinese	32 (37)	
	Caucasian	23 (26)	
	South Asian	9 (10)	
	Japanese	5 (6)	
	Southeast Asian	3 (3)	
	Other (eg, African, West Asian, Latin American, Indigenous, other)	15 (17)	
**Dog ownership, n (%)**			
	Yes	17 (20)	
	No	70 (80)	
**Employment status, n (%)**			
	Full time/part time	64 (74)	
	Other	23 (26)	
**Marital status, n (%)**			
	Married/common law	73 (84)	
	Other	14 (16)	
**Household members participating in intervention, n (%)**			
	One adult only	22 (25)	
	Multiple adults only	8 (9)	
	One adult and child(ren)	29 (33)	
	Multiple adults and child(ren)	28 (32)	
Age (years), mean (SD)			39.8 (7.4)
Number of children, mean (SD)			1.8 (1.1)

^a^CAD $1=US $0.79.

### Quantitative Findings

#### Changes in Physical Activity (Pooled Analysis)

Compared to that at baseline, the mean time spent walking at 8 weeks, but not 4 weeks, was significantly higher (*P*=.005) with no statistically significant differences found between baseline (T_0_) and the other time points (T_1_ and T_2_) for the other physical activity outcomes, including weekly minutes of MPA, VPA, and total physical activity, and frequency (days) of sufficient MVPA (Table 2).

**Table 2:** Differences in self-reported physical activity and sedentary behavior at baseline (T_0_), 4 weeks (T_1_), and 8 weeks (T_2_) (N=87).

Variable	Friedman test		T_0,_ mean (SD)	T_1_, mean (SD)	T_2_, mean (SD)	*P* value within-subject effect (time)^a^	
	*χ*^2^ (*df*=2)	*P* value				T_0_ vs T_1_	T_0_ vs T_2_
Walking (min/week)	9.394	.009	180.34 (262.92)	167.82 (155.48)	253.79 (315.23)	.21	.005
MPA^b^ (min/week)	1.465	.48	61.03 (86.76)	63.79 (97.55)	90.91 (158.56)	—	—
VPA^c^ (min/week)	0.469	.79	62.76 (73.62)	65.06 (82.41)	74.25 (111.74)	—	—
Total PA^d^ (MET^e^ min/week)	2.983	.23	1334.45 (1341.31)	1324.37 (1221.67)	1861.88 (2150.45)	—	—
Days of MVPA^f^≥30 min/day	1.979	.37	2.74 (1.98)	3.16 (2.04)	3.16 (2.00)	—	—
Sitting (min/day)	14.268	<.001	334.26 (201.64)	260.46 (185.15)	267.13 (190.07)	<.001	<.001
Screen time (min/day)	5.244	.07	175.86 (156.16)	144.71 (134.14)	145.40 (148.01)	—	—

^a^Wilcoxon signed-rank tests with *P*<.025 (planned comparisons T_0_ vs T_1_ and T_0_ vs T_1_) considered statistically significant. The Wilcoxon signed-rank test was only undertaken when the Friedman test was significant (*P*<.05).

^b^MPA: moderate-intensity physical activity.

^c^VPA: vigorous-intensity physical activity.

^d^PA: physical activity.

^e^MET: metabolic equivalent.

^f^MVPA: moderate-to-vigorous physical activity.

#### Changes in Physical Activity (Effect Modification)

None of the physical activity gain scores was significantly different between those with and without prior wearable tracker experience, suggesting a similar effect of the intervention for both groups. Time spent walking among those with prior wearable tracker experience was significantly (*P*=.03) different between the time points, but none of the planned comparisons reached significance (*P*>.025; Table 3).

A significant difference in walking gain scores was found between those with and without prior eHealth app experience (T_2_–T_0_, *P*=.04), suggesting that the effect of the intervention was different between the two groups. Compared to that at baseline, participants with no prior eHealth experience increased their minutes of walking at 8 weeks (*P*<.001) with no significant difference over time found among those with prior eHealth experience (Table 4).

We found a significant difference in walking gain scores between individuals from households with only adults versus households that included children participating in the program (T_2_–T_0_, *P*=.04), suggesting that the effect of the intervention was different between the two groups. Significant differences were found in walking between baseline and 4 weeks (*P*=.02) and 8 weeks (*P*<.001) only for individuals from households that included children participating in the program. We also found a significant increase in total physical activity at 8 weeks compared to baseline (*P*=.01) among this same group (Table 5). However, the total physical activity gain scores were not significantly different from T_2_ to T_0_ (*P*=.16), suggesting that the effect of the intervention was similar between individuals from households that included only adult participants and individuals from households that included child participants (Table 5).

**Table 3:** Differences in self-reported physical activity and sedentary behavior at baseline (T_0_), 4 weeks (T_1_), and 8 weeks (T_2_) according to history of activity tracker use (N=87).

Variable			Friedman test				T_0_, mean (SD)		T_1_, mean (SD)		T_2_, mean (SD)		*P* value within-subject effect (time)^a^		
			*χ*^2^ (*df*=2)		*P* value								T_0_ vs T_1_		T_0_ vs T_2_
**Prior use of activity tracker (n=40)**															
	Walking (min/week)	6.993		.03		186.00 (238.22)		168.50 (145.51)		208.75 (185.72)		.38		.07	
	MPA^b^ (min/week)	1.476		.48		62.5 (95.48)		67.25 (92.68)		76.50 (110.70)		—		—	
	VPA^c^ (min/week)	1.317		.52		69.00 (77.65)		72.5 (80.69)		78.5 (78.92)		—		—	
	Total PA^d^ (MET^e^ min/week)	4.088		.13		1398.80 (1269.45)		1384.05 (1165.64)		1614.88 (1369.12)		—		—	
	Days of MVPA^f^≥30 min/day	0.774		.68		2.70 (1.94)		3.25 (1.86)		3.37 (2.03)		—		—	
	Sitting (min/day)	11.036		.004		365.50 (203.61)		285.00 (182.01)		292.50 (192.80)		.002		.04	
	Screen time (min/day)	3.263		.20		195.25 (173.31)		154.25 (108.96)		162.25 (168.96)		—		—	
**Never used activity tracker (n=47)**															
	Walking (min/week)	3.012		.22		175.53 (284.74)		167.23 (165.03)		292.13 (391.49)		—		—	
	MPA (min/week)	4.114		.13		59.79 (79.63)		60.85 (102.42)		103.19 (190.47)		—		—	
	VPA (min/week)	0.013		.99		57.45 (70.42)		58.72 (84.20)		70.64 (134.28)		—		—	
	Total PA (MET min/week)	0.973		.62		1287.34 (1411.50)		1273.57 (1277.72)		2072.11 (2637.84)		—		—	
	Days of MVPA≥30 min/day	1.244		.54		2.79 (2.03)		3.08 (2.19)		2.98 (1.97)		—		—	
	Sitting (min/day)	4.333		.12		307.66 (198.22)		239.57 (187.16)		245.53 (187.06)		—		—	
	Screen time (min/day)	3.810		.15		159.36 (139.36)		136.60 (153.07)		131.06 (127.66)		—		—	

^a^Wilcoxon signed-rank tests with *P*<.025 (planned comparisons T_0_ vs T_1_ and T_0_ vs T_1_) considered statistically significant. The Wilcoxon signed-rank test was only undertaken when the Friedman test was significant (*P*<.05).

^b^MPA: moderate-intensity physical activity.

^c^VPA: vigorous-intensity physical activity.

^d^PA: physical activity.

^e^MET: metabolic equivalent.

^f^MVPA: moderate-to-vigorous physical activity.

**Table 4:** Differences in self-reported physical activity and sedentary behavior at baseline (T_0_), 4 weeks (T_1_), and 8 weeks (T_2_) according to history of eHealth app use (N=87).

Variable			Friedman test				T_0_, mean (SD)		T_1_, mean (SD)		T_2_, mean (SD)		*P* value within-subject effect (time)^a^		
			*χ*^2^ (*df*=2)		*P* value								T_0_ vs T_1_		T_0_ vs T_2_
**Prior use of eHealth app (n=43)**															
	Walking (min/week)	2.229		0.328		186.51 (225.17)		170.23 (150.92)		211.16 (287.61)		—		—	
	MPA^b^ (min/week)	0.043		0.979		68.37 (89.39)		65.35 (75.29)		99.77 (161.10)		—		—	
	VPA^c^ (min/week)	1.938		0.380		61.39 (76.67)		80.93 (95.74)		86.98 (137.52)		—		—	
	Total PA^d^ (MET^e^ min/week)	2.306		0.316		1408.05 (1231.73)		1408.28 (1110.01)		1842.88 (2428.60)		—		—	
	Days of MVPA^f^≥30 min/day	8.122		0.017		2.65 (1.91)		3.70 (2.08)		3.37 (2.08)		.001		.03	
	Sitting (min/day)	16.014		<0.001		352.56 (186.57)		241.86 (168.00)		278.60 (177.93)		<.001		.004	
	Screen time (min/day)	4.262		0.119		206.05 (188.28)		128.37 (102.91)		143.95 (142.20)		—		—	
**Never used eHealth app (n=44)**															
	Walking (min/week)	8.488		0.014		174.32 (297.76)		165.45 (161.48)		295.45 (338.15)		.32		<.001	
	MPA (min/week)	2.764		0.251		53.86 (84.53)		62.27 (116.16)		82.27 (157.40)		—		—	
	VPA (min/week)	1.048		0.592		64.09 (71.38)		49.55 (64.30)		61.82 (78.60)		—		—	
	Total PA (MET min/week)	4.409		0.110		1262.52 (1451.08)		1242.36 (1329.49)		1880.45 (1867.42)		—		—	
	Days of MVPA ≥30 min/day	1.248		0.536		2.84 (2.06)		2.64 (1.88)		2.95 (1.92)		—		—	
	Sitting (min/day)	1.987		0.370		316.36 (215.99)		278.64 (200.76)		255.91 (202.66)		—		—	
	Screen time (min/day)	1.822		0.402		146.36 (111.11)		160.68 (158.49)		146.82 (155.11)		—		—	

^a^Wilcoxon signed-rank tests with *P*<.025 (planned comparisons T_0_ vs T_1_ and T_0_ vs T_1_) considered statistically significant. The Wilcoxon signed-rank test was only undertaken when the Friedman test was significant (*P*<.05).

^b^MPA: moderate-intensity physical activity.

^c^VPA: vigorous-intensity physical activity.

^d^PA: physical activity.

^e^MET: metabolic equivalent.

^f^MVPA: moderate-to-vigorous physical activity.

**Table 5:** Differences in self-reported physical activity and sedentary behavior at baseline (T_0_), 4 weeks (T_1_), and 8 weeks (T_2_) according to level of household participation (N=87).

Variable		Friedman test			T_0_, mean (SD)		T_1_, mean (SD)	T_2_, mean (SD)		*P* value within-subject effect (time)^a^	
		*χ*^2^ (*df*=2)	*P* value						T_0_ vs T_1_		T_0_ vs T_2_
**Adults and children participating (n=57)**											
	Walking (min/week)	15.282	<0.001	128.07 (131.05)		164.74 (135.44)		251.40 (303.42)	.02		<.001
	MPA^b^ (min/week)	0.304	0.859	58.07 (83.23)		60.70 (87.64)		70.70 (94.38)	—		—
	VPA^c^ (min/week)	1.899	0.387	60.70 (68.27)		59.82 (79.97)		72.10 (81.96)	—		—
	Total PA^d^ (MET^e^ min/week)	5.982	0.050	1130.00 (999.47)		1268.54 (1074.55)		1683.67 (1693.29)	.33		.01
	Days of MVPA^f^≥30 min/day	4.383	0.112	2.58 (1.94)		3.17 (2.20)		3.25 (2.15)	—		—
	Sitting (min/day)	11.223	0.004	360.88 (203.15)		277.02 (199.61)		280.88 (191.29)	<.001		.002
	Screen time (min/day)	3.362	0.186	183.51 (176.85)		136.67 (137.05)		146.67 (157.96)	—		—
**Adults only participating (n=30)**											
	Walking (min/week)	0.241	0.887	279.67 (395.30)		173.67 (190.25)		258.33 (341.85)	—		—
	MPA (min/week)	2.960	0.228	66.67 (94.33)		69.67 (115.47)		129.33 (234.58)	—		—
	VPA (min/week)	0.526	0.769	66.67 (83.97)		75.00 (87.40)		78.33 (155.01)	—		—
	Total PA (MET min/week)	0.218	0.897	1722.90 (1779.90)		1430.43 (1475.97)		2200.50 (2827.74)	—		—
	Days of MVPA ≥30 min/day	0.587	0.746	3.07 (2.03)		3.13 (1.74)		3.00 (1.70)	—		—
	Sitting (min/day)	3.453	0.178	283.67 (191.93)		229.00 (152.14)		241.00 (188.14)	—		—
	Screen time (min/day)	2.272	0.321	161.33 (107.63)		160.00 (129.32)		143.00 (129.51)	—		—

^a^Wilcoxon signed-rank tests with *P*<.025 (planned comparisons T_0_ vs T_1_ and T_0_ vs T_1_) considered statistically significant. The Wilcoxon signed-rank test was only undertaken when the Friedman test was significant (*P*<.05).

^b^MPA: moderate-intensity physical activity.

^c^VPA: vigorous-intensity physical activity.

^d^PA: physical activity.

^e^MET: metabolic equivalent.

^f^MVPA: moderate-to-vigorous physical activity.

#### Changes in Sedentary Behavior (Pooled Analysis)

Compared to that at baseline, the mean time spent sitting was significantly lower at 4 weeks (*P*<.001) and 8 weeks (*P*<.001), respectively. However, there were no significant differences in daily screen time between time points (Table 2).

#### Changes in Sedentary Behavior (Effect Modification)

We found no significant differences in sitting or screen time gain scores between those with and without prior wearable tracker experience, suggesting a similar response to the intervention in both groups. Among participants with prior wearable tracker experience, time spent sitting was significantly lower at 4 weeks relative to that at baseline (*P*=.002) (Table 3).

Similarly, we found no significant differences in sitting or screen time gain scores between those with and without prior eHealth experience. However, compared to that at baseline, the mean time spent sitting was significantly lower at 4 weeks (*P*<.001) and 8 weeks (*P*=.004) among those with prior eHealth experience only (Table 4).

No significant differences were found in sitting or screen time gain scores between individuals from households with only adults versus households that included child program participants. Nevertheless, we found significant differences over time in sitting among those from households with child participants (T_0_ vs T_1_, *P*<.001; T_0_ vs T_2_, *P*=.002) (Table 5).

### Qualitative Findings

#### Overview

Three themes associated with behavior change in response to the VPS program emerged from the interviews: *Increased Physical Activity, Reduced Sedentary Behavior,* and *Other Health Benefits*. During interviews, participants described how the VPS program including the wearable activity tracker, eHealth dashboard, and Garmin Connect had supported their physical activity, sedentary behavior, and provided other health benefits during the 8-week intervention (Textbox 1). Saturation was obtained, with responses emerging from interviews often being repeated by different participants.

**Themes, subthemes, and representative quotes reflecting participants’ experiences during the Vivo Play Scientist program.**

**Increased physical activity**

***Changes in awareness, motivation, and behavior***

- “I’m paying attention to how many steps I take and if I took a certain amount each day. I’ve even included [walking] instead of taking my 30-minute lunch break.” [female, 41 years]
- “I’ll set goals for myself quietly that no one knows about. I’m accountable to myself if I don’t do it. [It’s] a way to motivate myself.” [female, 43 years]
- “This is a physical motivator for me because I look at it, it’s like, ‘Oh crap. I didn’t get my step count’.” [female, 54 years]
- “I know how active I am, it [the VScore] shows on the graph, as a family graph and individual. It gives you an indication of how you are doing.” [male, 36 years]

***Negative impacts of using wearable technology***

- “I stopped using a fitness tracker, was because I was finding it was taking the enjoyment out of exercise. Because I was getting too focused on how I compared to other people.” [female, 36 years]

***Changes in family physical activity***

- “It’s worked really well for my son, because it got him to actually want to walk more. He wants to be more active because he wants to get his steps in. He wants to get a badge.” [female, 52 years]
- “It’s been really positive, especially with COVID. Because we were pretty housebound, the kids haven’t been able to go and play with their friends and stuff like that. It’s been a good incentive to go out and just explore and get active.” [female, 39 years]
- “We’re looking at the average of our household, how we’re doing as overall health. As a family, this [the dashboard] is really helping us to understand, ‘Hey, what can we do together next time on a weekend?’ That helped us with our planning our activities together. So we were planning a little bit more what kind of activities we can try at home to do it together or taking turns doing it.” [female, 39 years]
- “I’ll say to my wife…‘What’s up with your VScore being on a 50, whereas even of our children are hovering around the high 60s low 70s and myself in the mid-70s at any given time?’ Right? I’ll say well then we as a family collectively have to help mom or help my son make up for that dip in the following weeks. I’ll just simply tell the family during dinnertime like ‘Someone scored low, we won’t mention who. Someone better start moving’.” [male, 41 years]

**Reduced sedentary behavior**

- “Because my work is mostly like 8 hours work, and I’m sitting all the time…when I get these beeps I actually will move. So it gives me a chance to take breaks too.” [male, 36 years]
- “With the Garmin watch on, I would feel more inclined to maybe just take a break from my studying or from my work and go and play with them for 5 or 10 minutes.” [female, 23 years]
- “I think as a whole, all those features, like track your steps every day, the dashboard and the challenges, the badges, which actually give you a little push to do it every day. The nudges that it gives you, that you need to move, they all help as a whole to motivate you to be more active.” [male, 36 years]

**Other health benefits**

***Enhanced mental well-being***

- “I found that if I didn’t do enough activity, my emotional state was worse. If I have higher, more activity, more like if I’m running or doing just more, higher intensity workouts, it’s for me, I have a better day. My whole mood is much, much better.” [female, 52 years]

***Health education opportunity for children***

- “It’s a matter of encouraging something that I would hope that they would keep as a lifestyle thing…keeping in mind that I am raising kids that will someday be adults and hopefully to transmit to them a mentality that includes physical fitness in their lives.” [female, 41 years]

#### Increased Physical Activity

For many participants, using the wearable activity tracker and dashboard increased awareness of their own physical activity, which motivated them to improve their behavior. Many participants described how the step count displayed on the wearable tracker motivated them to be physically active and how they used this information as a benchmark with which to compare their personal goals and progress. Few participants described how the dashboard and vScore supported their physical activity. Those who participated in the program with other household members, including with children, described how the program supported increases in their personal physical activity via spending more time being active as a family. Families who participated together in the program used their tracked steps and vScore to hold members accountable to improving their personal and family’s overall physical activity levels. However, for some participants, especially those who were already active or who experienced physical barriers (eg, poor weather and facility closures due to the COVID-19 pandemic) described the program as having little impact on their physical activity. Some participants even commented that certain aspects of the program such as the comparison of activity levels with others detracted from the enjoyment of undertaking physical activity.

#### Reduced Sedentary Behavior

Similar to physical activity, participants described how the program, and notably the wearable tracker, had increased awareness about their own sedentary behavior (Textbox 1). Participants commented that “move” prompts or notifications from the wearable tracker, as well as just wearing the device, encouraged them to break up periods of sedentary behavior such as sitting with movement activity and “nudged” them to be active.

#### Other Health Benefits

While not the main aim of the VPS program, participants perceived that their mental as well as physical and social health had improved as a result of participating (Textbox 1). Several participants recognized that their physical activity positively contributed to their sense of mental well-being, stress level, and sleep quality. For example, one participant described how her mood and mental state appeared to be related to her physical activity levels. Some participants noticed reductions in their weight, while others enjoyed the increased family interactions that resulted from participating as a household in the program. A few participants also perceived the program as an opportunity to educate their children about the importance of physical activity, fitness, and health.

## Discussion

### Principal Results

The aim of this study was to evaluate the effectiveness of a community-focused physical activity intervention designed and implemented by a local recreational facility that incorporated wearable and eHealth technology. Congruent with previous evidence [14-17], the quantitative and qualitative findings from our 8-week evaluation suggest that providing participants with a free-of-cost wearable activity tracker (Vivofit4) and access to a customized eHealth dashboard has the potential to both improve physical activity via increases in walking and to reduce sedentary behavior via discouraging sitting. Specifically, during the 8-week intervention, participants, on average, increased their walking time by approximately 73 minutes/week and reduced their sitting time by approximately 67 minutes/day. An increase in walking of this magnitude has the potential to protect against all-cause and cardiovascular-related mortality and chronic disease [48-50]. Given that a 60-minute increase in sitting time has been found to be associated with an increased risk of cardiovascular disease (4%), cancer (1%), and all-cause mortality (1%) [51], this reduction in sitting time also has clinical relevance. Despite increases in the total physical activity, MPA, VPA, and MVPA, and decreases in screen time during the intervention, none of these changes reached statistical significance. Nevertheless, participants described that wearing Vivofit4 and accessing the dashboard motivated them to monitor and modify their physical activity and sedentary behavior. These findings highlight the usefulness of wearable technology and eHealth apps in supporting physical activity behavior change in the short term [16,17,23-26]. Importantly, the VPS program appeared to be effective despite being implemented under the COVID-19 public health restrictions.

Our evaluation of the VPS program only included adults; however, Vivo offered the program to individuals and families. The program design was relatively minimalistic. Apart from offering participants with a free-of-cost commercially available wearable tracker and access to the customized eHealth dashboard, the program included no other formal intervention components (eg, no health-promotion messages or reminders such as push notifications, exercise classes, counseling sessions, or group activities) to encourage behavior change. The use and application of feedback from Vivofit4 and the dashboard was self-determined by participants, and participants received no advice as to *how* or by *how much* they should modify their behaviors. Notably, the frequency of accessing the eHealth dashboard among our participants was low (approximately 1 day/week), which is consistent with a previous study using a similar dashboard provided by Vivametrica [36]. However, allowing multiple members from the same household to participate in the VPS program may have had an unintended positive consequence on the effectiveness of the program for some individuals. During interviews, individuals described how participating in the program as a family or with other household members (especially with children) encouraged changes in physical activity and sedentary behavior. This finding aligns with previous evidence suggesting that the social environment can influence physical activity in adults [52,53] and children [54,55]. Participants described the VPS program as providing household members with opportunities to increase physical activity via competition, sharing of behavioral data, developing shared behavioral goals (eg, contributing to a household averaged vScore), motivation, and opportunities to help other household members achieve their personal behavior goals. Our quantitative findings demonstrated that adults from households that included children participating in the VPS program significantly increased their walking by approximately 123 minutes/week and their total physical activity by 553 MET-minutes/week, and decreased their sitting time by 80 minutes/day. Congruent with our findings, Schoeppe et al [56] observed an increase of 45 minutes/day among children and 26 minutes/day among parents in self-reported MVPA during a 6-week multicomponent family-centered intervention that included wearable trackers and family-focused physical activity strategies (eg, family challenges and leader boards). Parental support (eg, motivating and educating), behavior modeling, and shared activities are important for encouraging physical activity in children [55,57,58]. Our findings suggest that interventions that include wearable and eHealth technology together with strategies that can encourage family engagement may be beneficial for improving physical activity and even sedentary behavior among adults.

Participants with and without prior wearable tracker experience had a similar response to the VPS program in terms of changes in physical activity and sedentary behavior. Our data did not allow us to differentiate among those with prior experience into current and former wearable tracker users. Other studies have found differences in current physical activity levels, perception of influence on physical activity, sociodemographic characteristics, health conditions, length of wearable tracker use, and reasons for using wearable trackers between current and former users [59-61]. Importantly, our findings suggest that the effectiveness of the program appeared independent of prior wearable tracker experience, at least in the short term. However, the novelty of the eHealth dashboard might have had a positive impact on behavior [62]. Participants with no prior eHealth experience reported increases in weekly walking during the 8-week intervention, whereas no changes were observed in those with prior eHealth experience. There is no clear explanation for this finding. Speculatively, those with prior eHealth experience may have been less sensitive to the changes observed in the vScore or possibly they were already using preferred alternative eHealth apps. Adults who positively assess an eHealth app and are resolute in achieving their health goals may be more likely to continue using the app, whereas those who negatively assess an eHealth app but are determined to achieve their health goals may be more likely to switch to another app [63].

While the aim of the VPS program was to increase physical activity and decrease sedentary behavior, participants experienced other benefits. In addition to being more cognizant of their behavior patterns, some participants became more aware of how their levels of physical activity were associated with their sense of well-being and mood. Others derived benefits in terms of improved sleep and perceived reductions in weight. Elsewhere, people reported perceived changes in their sleeping and eating patterns due to using wearable trackers [60]. Moreover, reductions in weight and lipid profiles, and perceived increases in well-being have been found among older adults with chronic medical conditions after 12 weeks of receiving a free wearable tracker [64]. Our results are also supported by findings elsewhere suggesting that in addition to higher physical activity (MPA, walking, and total physical activity) and less sedentary time, current users of commercially available wearable trackers report better sleep quality and quality of life than nonusers [65]. Interventions involving wearable and eHealth technology should consider measuring a range of physical and mental health outcomes, in addition to physical activity and sedentary behavior, to better understand the potential effects on overall health and well-being.

### Limitations

Wearable trackers and eHealth apps range in the functions they offer and the ways in which they encourage or support behavior change (eg, movement prompts, behavior goal-setting, goal achievement notifications, data sharing) [66]. Thus, the effects of Vivofit4 and the vScore on physical activity and sedentary behavior found in our study may not generalize to other wearable or eHealth technologies. Nevertheless, our findings tend to be aligned with previous evidence suggesting that wearable activity trackers support improvements in physical activity and sedentary behavior [16,17,23-26]. Participants volunteered for the program and therefore may reflect a highly motivated and healthy population, limiting generalizability. Desires to maintain or improve fitness, weight, and quality of life are motivating factors associated with the use of fitness applications [62]. Moreover, the effectiveness of the VPS program for improving walking and sitting may have been amplified due to participants having more time or seeking opportunities to undertake physical activity during the COVID-19 pandemic public health restrictions. Notably, only about half of the eligible individuals who registered for the program participated in the evaluation. Individuals registered but who did not participate in the study may have been less motivated to be physically active, knowing that their behavior would be monitored by our team. Moreover, we cannot rule out Hawthorn bias, whereby some participants may have been more inclined to use the wearable tracker and change their behavior knowing that their physical activity, sedentary behavior, and level of use of the device would be measured over the course of the program.

Our single-arm study design did not include a control group that would have allowed competing explanations of our findings to be eliminated (eg, self-selection, history, statistical regression, experimental mortality). However, the inclusion of qualitative data provided explanations in support of our quantitative results, thus adding robustness to our findings. Despite the shortcomings of our quasiexperiment, our findings support the short-term increases in physical activity and steps consistently found by randomized controlled trials of interventions using wearable trackers alone or in combination with other components [67]. We did not have access to device and dashboard data; thus, we relied on self-report data collected using questionnaires. Self-report measures are known to provide bias estimates of physical activity [68]. We are not able to generalize our findings beyond the 8-week observation period. In previous studies, former wearable tracker users reported wearing their devices for only about 5 months [59,60]. This may suggest that other intervention components (eg, check-ins, reminder emails or telephone calls, support groups) would be needed to support adherence in using wearable trackers during longer interventions. The effects of using wearable and eHealth technology on physical activity and sedentary behavior over the long term require further investigation.

### Conclusions

The use of wearable activity trackers and eHealth apps may lead to improvements in walking and reduction in sitting time, and could enhance physical and mental well-being. Importantly, the VPS program demonstrated the potential to positively influence physical activity in the short term despite the challenge of being implemented under COVID-19 public health restrictions. Implementation of physical activity interventions that include wearable and eHealth technology could be an option for recreational facilities as they face challenges in delivering recreational programming to families during pandemic lockdowns and capacity restrictions. Future research may consider investigating the cost-effectiveness and sustainability of providing free-of-cost wearable and eHealth technology to participants as a stand-alone intervention for increasing physical activity and reducing sedentary behavior. Moreover, future studies are needed to determine which populations may derive the most benefit from stand-alone interventions that provide free-of-cost wearable and eHealth technology. Recreational facilities that provide free-of-cost commercially available wearable trackers together with customized eHealth apps as part of community-focused health-promotion interventions have the potential to support increases in physical activity and reduce sedentary behavior in the short term.

## Abbreviations

IPAQ-SF: International Physical Activity Questionnaire Short-Form
MET: metabolic equivalent
MPA: moderate-intensity physical activity
MVPA: Moderate-to-vigorous physical activity
VPA: vigorous-intensity physical activity
VSP: Vivo Play Scientist Program
